# Supplementary material for: Isolation and Characterization of Plant-Growth-Promoting, Drought-Tolerant Rhizobacteria for Improved Maize Productivity
Source: Plants (Basel). 2024 May 8;13(10):1298. doi: 10.3390/plants13101298 (PMC11125291; doi:10.3390/plants13101298)
Supplement: Supplementary file 1 [file plants-13-01298-s001.zip › plants-2738885-supplementary.pdf]

Isolation and Characterization of Plant-Growth-Promoting, Drought Tolerant Rhizobacteria for Improved Maize Productivity

Victor Funso Agunbiade, Ayomide Emmanuel Fadiji, Nadège Adoukè Agbodjato and Olubukola Oluranti Babalola *

Food Security and Safety Focus Area, Faculty of Natural and Agricultural Sciences, North-West University, Mmabatho, South Africa

* Correspondence: Olubukola Oluranti Babalola; E-mail: olubukola.babalola@nwu.ac.za; Tel.: +27-18-389-2568

Supplementary files

**Parameters**	**Rhizobacterial Isolates**					
**Isolate code**	**A5-1**	**A1-2**	**B8-3**	**B12-4**	**B9-5**	**B15-6**
**Identification of isolates according to NCBI**	*B. licheniformis*	*A. caviae*	*B. cereus*	*P. flexa*	*B. licheniformis*	*B. simplex*
**Microscopic appearance**	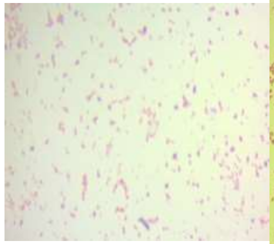	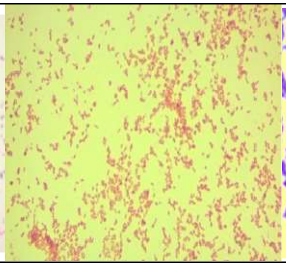	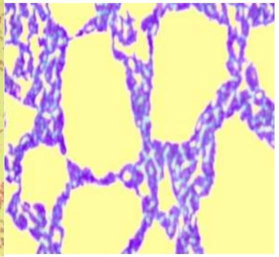	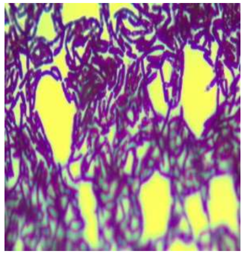	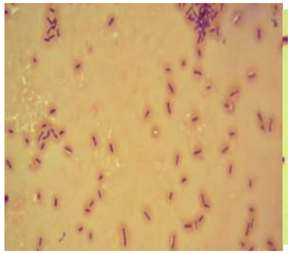	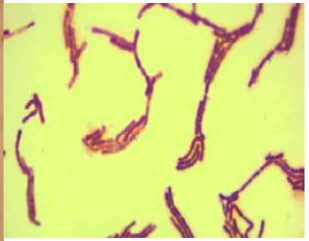
**Accession No :**	ON745408	ON745409	ON745413	ON745414	ON745412	ON745413
**% Similarity**	100	99.83	100	100	100	100
**Isolate code**	**C6-7**	**C7_8**	**C1-9**	**A9-10**	**A10-11**	
**Identification of isolates according to NCBI**	*P. flexa*	*A. veronii*	*P. aryabhattai*	*B. halotolerans*	*B. endophyticus*	

**Microscopic appearance**	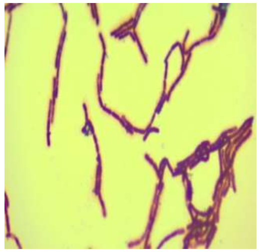	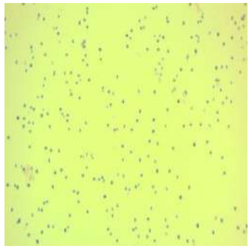	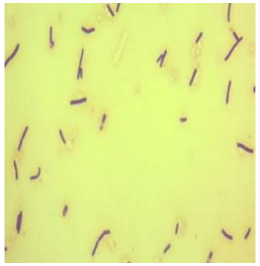	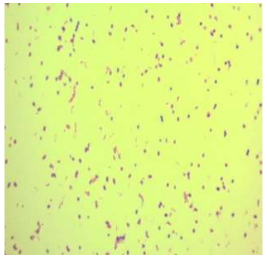	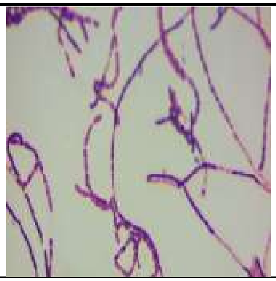
**Accession No :**	ON745414	ON745415	ON745416	ON745417	ON745418
**% Similarity**	100	100	100	100	100

Figure S1. Microscopic View of Rhizobacterial Strains Isolated from Maize Plants.

Table S1. Morpho-biochemical Characteristics of The Isolated Rhizobacterial Strains.

Bacteria identified	Gram Reaction	Shape	Motility	Catalase	Oxidase	SH	CIT	NR	MAL	LAC	GLU	SUC	FRU	XYL	GAL
A5-1	+	Short rod	Motile	+	+	-	+	+	+	-	+	+	+	+	+
A1-2	-	Short rod	Motile	+	+	+	+	+	+	+	+	+	-	-	-
B8-3	+	Short rod	Motile	-	+	+	+	+	+	-	+	+	-	-	-
B12-4	+	Rod	Motile	+	-	-	+	ND	+	+	+	+	-	-	+
B9-5	+	Rod	Motile	+	+	+	-	+	+	+	+	+	-	-	-
B15-6	+	Rod	Motile	-	+	-	-	+	+	+	+	+	-	-	-
C6-7	+	Bacilli	Motile	+	-	+	+	+	+	-	+	+	+	+	+
C7_8	-	Cocci	Motile	+	+	+	+	+	+	+	+	+	-	-	-
C1-9	+	Rod	Motile	+	-	-	+	+	+	-	+	+	-	-	-
A9-10	+	Cocci	Non-motile	+	-	-	+	+	+	-	+	+	+	ND	-
A10-11	+	Rod	Non-motile	+	+	-	+	+	+	-	+	+	-	-	+

Legend: - = negative, + = positive, ND=Not determined, SH=Starch Hydrolysis, CIT=Citrate, NR=Nitrate Reduction, MAL=Maltose, SUC=Sucrose, GLU=Glucose, LAC=Lactose, FRU=Fructose, XYL= Xylose, GAL=Galactose.

The calculation of Field Capacity (FC)
Add 6 kg of soil 3 Liters of Maximum Water Rentention Capapcity (MRC), i.e. 50 ml MRC water for 100 g of soil.
2/9 MRC water was used. i.e. (3000 ml x 2)/9 = 666, 66 ml or 670 ml.

For drought:
100%, 670 ml of water for 6 kg of soil was used.
50%, 335 ml of water for 6 kg of soil was used.
25%, 167 ml, 5, or 170 ml of water for 6 kg of soil was used.
